# Sampling at community level by using satellite imagery and geographical analysis

**DOI:** 10.2471/BLT.14.140756

**Published:** 2014-06-17

**Authors:** Veronica Escamilla, Michael Emch, Leonard Dandalo, William C Miller, Francis Martinson, Irving Hoffman

**Affiliations:** a Institute for Global Health and Infectious Diseases, University of North Carolina-Chapel Hill, 130 Mason Farm Road, Chapel Hill, NC 27599, United States of America (USA).; b Department of Geography, University of North Carolina-Chapel Hill, Chapel Hill, USA.; c University of North Carolina Project, Lilongwe, Malawi.; d Department of Medicine, University of North Carolina-Chapel Hill, Chapel Hill, USA.

## Abstract

**Problem:**

Traditional random sampling at community level requires a list of every individual household that can be randomly selected in the study community. The longitudinal demographic surveillance systems often used as sampling frames are difficult to create in many resource-poor settings.

**Approach:**

We used Google Earth imagery and geographical analysis software to develop a sampling frame. Every household structure within the catchment area was digitized and assigned coordinates. A random sample was then generated from the list of households.

**Local setting:**

The sampling took place in Lilongwe, Malawi and formed a part of an investigation of the intensity of *Plasmodium falciparum* transmission in a multi-site Phase III trial of a candidate malaria vaccine.

**Relevant changes:**

Creation of a complete list of household coordinates within the catchment area allowed us to generate a random sample representative of the population. Once the coordinates of the households in that sample had been entered into the hand-held receivers of a global positioning system device, the households could be accurately identified on the ground and approached.

**Lessons learnt:**

In the development of a geographical sampling frame, the use of Google Earth satellite imagery and geographical software appeared to be an efficient alternative to the use of a demographic surveillance system. The use of a complete list of household coordinates reduced the time needed to locate households in the random sample. Our approach to generate a sampling frame is accurate, has utility beyond morbidity studies and appears to be a cost-effective option in resource-poor settings.

## Résumé

**Problème:**

L'échantillonnage aléatoire traditionnel au niveau de la communauté requiert une liste de tous les ménages individuels qui peuvent être choisis aléatoirement dans une étude communautaire. Les systèmes de surveillance démographique longitudinaux, qui sont souvent utilisés comme base d'échantillonnage, sont difficiles à créer dans de nombreux endroits à faibles ressources.

**Approche:**

Nous avons utilisé l'imagerie de Google Earth et un logiciel d'analyse géographique pour développer une base d'échantillonnage. Chaque structure de ménage dans la circonscription a été numérisée et associée à des coordonnées. Un échantillon aléatoire a ensuite été généré à partir de la liste des ménages.

**Environnement local:**

L'échantillonnage a été effectué à Lilongwe, au Malawi, et entrait ans le cadre d'une étude sur l'intensité de la transmission du *Plasmodium falciparum* dans un essai clinique multicentrique de phase III d'un vaccin contre le paludisme potentiel.

**Changements significatifs:**

La création d'une liste complète des coordonnées des ménages dans la circonscription nous a permis de générer un échantillon aléatoire représentatif de la population. Une fois que les coordonnées des ménages de cet échantillon ont été entrées dans les récepteurs portables d'un dispositif du système de positionnement universel, les ménages ont pu être précisément identifiés et approchés sur le terrain.

**Leçons tirées:**

Dans le développement d'une base d'échantillonnage géographique, l'utilisation de l'imagerie satellite de Google Earth et d'un logiciel géographique semblait être une alternative efficace à l'utilisation d'un système de surveillance démographique. L'utilisation d'une liste complète de coordonnées des ménages a réduit le temps nécessaire pour localiser les ménages dans un échantillon aléatoire. Notre approche de génération d'une base d'échantillonnage est précise, utile au-delà des études de morbidité et semble être une option économique dans les endroits à faibles ressources.

## Resumen

**Situación:**

El muestreo aleatorio tradicional a nivel comunitario requiere una lista de todos los hogares individuales que pueden ser seleccionados al azar en la comunidad de estudio. Los sistemas de vigilancia demográfica longitudinales que se utilizan a menudo como marcos de muestreo resultan difíciles de crear en muchos entornos con pocos recursos.

**Enfoque:**

Empleamos imágenes de Google Earth y un software de análisis geográfico para desarrollar un marco de muestreo. Se digitalizó y asignaron coordenadas a cada estructura familiar dentro de la zona de captación, y luego se generó una muestra al azar a partir de la lista de los hogares.

**Marco regional:**

El muestreo se llevó a cabo en Lilongwe (Malawi) y formó parte de una investigación sobre la intensidad de la transmisión del *Plasmodium falciparum* en un ensayo de fase III multicéntrico de una posible vacuna contra la malaria.

**Cambios importantes:**

La creación de una lista completa de las coordenadas de los hogares dentro del área de influencia nos ha permitido generar una muestra aleatoria representativa de la población. Una vez introducidas las coordenadas de los hogares en la muestra en los receptores portátiles de un dispositivo con un sistema de posicionamiento global, se pudo identificar con precisión a los hogares y aproximarse a estos en el terreno.

**Lecciones aprendidas:**

En el desarrollo de un marco de muestreo geográfico, el uso de imágenes de satélite de Google Earth y un software geográfico resultó ser una alternativa eficaz a la utilización de un sistema de vigilancia demográfica. La utilización de una lista completa de las coordenadas de los hogares reduce el tiempo necesario para localizar estos en la muestra aleatoria. Nuestro enfoque para generar un marco de muestreo es exacto, tiene una utilidad más allá de los estudios de morbilidad y parece ser una opción rentable en los entornos con recursos escasos.

## ملخص

**المشكلة:**

تتطلب العينات العشوائية التقليدية على صعيد المجتمع قائمة بكل أسرة فردية يمكن اختيارها عشوائياً في المجتمع محل الدراسة. ويصعب إنشاء نظم الترصد الديمغرافية الطولية التي تستخدم غالباً كإطارات لأخذ العينات في العديد من المناطق فقيرة الموارد.

**الأسلوب:**

قمنا باستخدام برنامج التصوير والتحليل الجغرافي Google Earth لوضع إطار لأخذ العينات. وتم تحويل كل هيكل أسري داخل منطقة المستجمع إلى الصيغة الرقمية وتعيين إحداثيات له. وبعد ذلك، تم تحديد عينة عشوائية من قائمة الأسر.

**المواقع المحلية:**

تم تنفيذ أخذ العينات في ليلونغوي، بملاوي وشكل جزءاً من تحري شدة سريان المتصورات المنجلية في تجربة متعددة المواقع من المرحلة الثالثة للقاح مرشح للملاريا.

**التغيّرات ذات الصلة سمح:**

لنا إنشاء قائمة كاملة بإحداثيات الأسر داخل منطقة المستجمع بتحديد عينة عشوائية ممثلة للسكان. وبمجرد إدخال إحداثيات الأسر في تلك العينة في أجهزة الاستقبال المحمولة يدوياً لجهاز النظام العالمي لتحديد المواقع، كان من الممكن تحديد الأسر بدقة على سطح الأرض والاقتراب منها.

**الدروس المستفادة:**

اتضح أن استخدام برنامج تصوير المواقع الجغرافية بالأقمار الاصطناعية Google Earth أثناء وضع إطار لأخذ العينات الجغرافية بديل فعال لاستخدام نظام الترصد الديمغرافي. وأدى استخدام قائمة كاملة بإحداثيات الأسر إلى تقليل الوقت اللازم لتحديد مواقع الأسر في العينة العشوائية. ويتميز أسلوبنا في وضع إطار لأخذ العينات بالدقة، كما أن فائدته تتعدى دراسات المراضة ويبدو أنه خيار عالي المردود في المناطق فقيرة الموارد.

## 摘要

**问题:**

传统的社区层面随机抽样需要可以在研究社区随机选择的各个家庭的列表。许多资源匮乏的地区难以创建通常作为抽样框架使用的纵向人口监测系统。

**方法:**

我们使用谷歌地球(Google Earth)图像和地理分析软件开发抽样框架。将来源区内每个家庭结构进行数字化并指定坐标。然后从家庭的列表生成随机样本。

**当地状况:**

抽样工作在马拉维利隆圭进行,是一项多点III期候选疟疾疫苗试验中恶性疟原虫传播强度调查的一部分。

**相关变化:**

建立来源区家庭坐标的完整列表可允许我们生成人口的随机样本代表。将样本中的家庭坐标输入到全球定位系统设备的手持接收机之后,就可以精确确定并接近地面上的家庭。

**经验教训:**

在开发地理抽样框架过程中,使用Google Earth的卫星图像和地理软件似乎是使用人口监测系统的有效替代方法。使用完整的家庭坐标列表可减少随机采样中寻找家庭所需的时间。我们生成抽样框架的方法非常准确,在发病率研究之外也可发挥效用,看来是资源贫乏环境中具有成本效益的选择。

## Резюме

**Проблема:**

Для традиционной случайной выборки на уровне общин требуется список всех отдельных домохозяйств, которые могут быть случайно выбраны в исследуемой общине. Системы продольного демографического надзора, которые часто используются в качестве основ выборки, сложно создаются во многих местах с ограниченными ресурсами.

**Подход:**

Для разработки основы выборки использовалась программа построения изображений и географического анализа Google Earth. Структура каждого домохозяйства в пределах района охвата была оцифрована с присвоением координат. Затем из списка домашних хозяйств была сформирована случайная выборка.

**Местные условия:**

Выборка производилась в Лилонгве, Малави, и являлась частью исследования интенсивности передачи *Plasmodium falciparum в многоцентровом испытании фазы 3 *экспериментальной вакцины от малярии.

**Осуществленные перемены:**

Создание полного списка координат домохозяйств в пределах района охвата позволило сгенерировать случайную репрезентативную выборку населения. После того как координаты домохозяйств этой выборки были введены в портативные приемники устройств системы глобального позиционирования, домохозяйства могут быть точно распознаны на месте и включены в исследования.

**Выводы:**

При разработке основы географической выборки использование программы построения изображений и географического анализа Google Earth, по-видимому, является эффективной альтернативой использованию системы демографического надзора. Использование полного списка координат домохозяйств сокращает время, необходимое для определения местоположения домохозяйств из случайной выборки. Наш подход к формированию основы выборки является точным, полезным не только для исследований заболеваемости, а также, по-видимому, является экономически эффективным вариантом в условиях ограниченности ресурсов.

## Problem

In estimating disease prevalence at community level, a sampling frame is required to ensure that the study sample is representative of the study community. Such a sampling frame typically lists all households that can be randomly selected in the study community and is often based on an existing demographic surveillance system. In the absence of such a system, one option is to create a complete listing of all households in the study area. In many malaria indicator surveys, for example, each household in the study area is visited, information on each head of household is captured and a sketch map showing the location of each household structure is drawn. However, this approach carries substantial field costs and these costs may increase when large census enumeration areas have to be sampled and a single segment has to be randomly selected from each enumeration area. Many resource-poor settings lack the funds and labour needed to create a full household listing in this manner – or to create and maintain a longitudinal demographic surveillance system.

Open-source software packages for geographical analysis are being increasingly employed in public health research, are available to those working in resource-poor settings and can be used to create an affordable sampling frame. We therefore used Google Earth and other geographical analysis software to generate an inexpensive sampling frame for a study on the intensity of *Plasmodium falciparum* transmission in Lilongwe, Malawi. In this paper, we report our experiences in constructing this sampling frame and using it to generate a random sample of households at the community level.

## Local setting

Malaria transmission intensity – which is dependent on several factors, including the parasite, vector, human host, environment and treatment – serves as an important guide for interventions of malaria control. The effects of such interventions on malaria morbidity may vary with transmission intensity.^1^ Thus, there is a need to assess not only how decreasing parasite exposure – via vaccines and other interventions – will alter malaria transmission but also how varying levels of malaria intensity may alter the efficacy of any intervention.

Lilongwe, Malawi, is one of 11 sites of a Phase III efficacy trial for the candidate malaria vaccine, RTS,S/AS01 (GlaxoSmithKline, Brentford, England). As part of this trial, temporal trends in the prevalence of human infection with *P. falciparum* in the vaccine catchment area needed to be assessed. The Lilongwe site is not covered by a demographic surveillance system and does not have the resources needed to conduct a complete field-based listing of all of the households in the catchment area. We therefore needed an alternative, inexpensive and low-resource approach to create a valid sampling frame for the investigation of malaria transmission intensity.

## Approach

We used two open-source software packages – Google Earth 5.1 (Google, Mountain View, United States of America) and Digipoint 2 (Zonum Solutions, Tucson, USA)^2^ – to generate a listing that showed the geographical location of each household. We then used two more programs – ArcGIS 9.3 (Environmental Systems Research Institute, Redlands, USA) and Hawth’s Tools for ArcGIS^3^ – to select a random sample from the complete geographical listing. Hand-held Garmin eTrex 10 global positioning system receivers (Garmin Ltd, Schaffhausen, Switzerland) were subsequently employed to find the sample households in the field.

We first identified the 61 census enumeration areas that defined the catchment area for the Lilongwe vaccine trial. For this, we used the enumeration areas of residence for the malaria patients attending the Malawi Ministry of Health’s area 18 health centre. The boundary files for the enumeration areas used in the 2008 census were obtained from the Malawi National Statistics Office. Community health workers used Garmin eTrex 10 receivers to collect the coordinates of each village within the catchment area. These coordinates and the boundary files were then imported into ArcGIS 9.3, converted into keyhole markup language (KML) files – for compatibility with Google Earth – and then imported into Google Earth so that the boundary of the catchment area could be defined. Village locations were overlaid onto the boundaries of enumeration areas and enumeration areas that included household structures within villages were digitized.

**Fig. 1:**
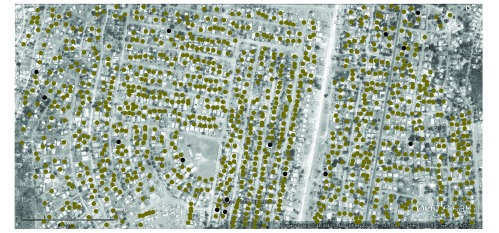
Zoomed Google Earth image of the sampling frame, Lilongwe, Malawi, 2010

Note: The random sample of households to be surveyed are indicated by black dots. The complete list of households is indicated by green dots. The full length of the scale bar – which formed part of the image downloaded from Google Earth – represents a distance of 288 m.

After the catchment area was defined in Google Earth, a list of the coordinates of every household structure in the area was generated. Individual household structures on the Google Earth satellite images of the catchment area were digitized using Digipoint 2 rather than Google Earth because this simplified the saving of multiple digitized points. The satellite images that we used had been recently updated – on 11 December 2009 or 4 June 2010 – and had sufficient resolution to identify individual structures (Fig. 1). However, they could not be used to distinguish houses from local commercial structures that were similar to the local houses in terms of size and construction material.

Map data: Google, digital globe.

All of the digitized points – approximately 18 000 – were converted to Universal Transverse Mercator coordinates in Digipoint 2 and then imported into ArcGIS 9.3. We then used the random selection tool from Hawth’s Tools for ArcGIS to generate a simple random sample of 880 household structures. The size of the sample required had already been defined by the manufacturer of the candidate vaccine. The coordinates of the structures in the random sample were entered into Garmin eTrex 10 receivers so that health workers given the receivers could find and visit the structures. Households were visited in the order of their random selection. In instances where two houses were very closely situated, the house closest to the relevant coordinates was selected. Structures that were included in the initial sample but found to be latrines, kitchens or commercial structures when visited were noted but otherwise ignored, and not replaced by new resampled coordinates. Houses found to be unoccupied or occupied only by children when first visited were revisited at a later date. If an adult was present in a visited household, demographic information was collected and the household members were invited to participate in our malaria survey at a later date.

## Evaluation and lessons learnt

By using Google Earth satellite imagery and geographical methods we were able to create a geographical sampling frame and select a representative random sample of the target population for our study. We eventually evaluated *P. falciparum* transmission during peak season – February to June – in 2011, 2012 and 2013. For each annual survey, we resampled the complete listing of household coordinates. Households that were investigated more than once were easily identified using the spatial join tool within ArcGIS 9.3.

Our method of generating a geographical household list appeared efficient and cost-effective and required relatively few resources other than the Garmin eTrex 10 receivers and an ArcGIS software licence. The digitizing exercise was completed by one research assistant within 3 months.

The geographical listing of households was found to be fairly accurate when used by community health workers on the ground. Fewer than 5.0% of the structures included in our initial list of structures to be visited were found to be latrines, kitchens or commercial structures. The ratio of male to female subjects in our sample – 1.08 – was very similar to that recorded in the Malawi demographic and health survey in 2010 –1.07.^4^

The use of Google Earth imagery and geographical methods to assemble a household list appears to be an efficient use of time in the field. Households that were targeted for study could be rapidly located on the ground by a community health worker with a global positioning system receiver. The list could be easily updated and could provide a useful framework for the future development of a demographic surveillance system. All survey data could be linked to household coordinates and displayed in ArcGIS – allowing spatial patterns in any household characteristic to be illustrated and investigated. Using our method of generating a sampling frame, all households in a target area can be digitized relatively easily, regardless of the area’s size. There is no need to segment large study areas before sampling. In the future, it may be possible to distinguish households from industrial buildings, even in densely populated areas, by using high-resolution satellite or aerial imagery,^5^^,^^6^ although identification of the use of multi-storey buildings from such imagery is likely to remain a challenge.

Our method of generating a sampling frame could be used for many health investigations. Google Earth has already been employed to identify and sample household clusters,^7^^,^^8^ capture neighbourhood characteristics that influence health outcomes^9^ and improve disease surveillance.^5^^,^^6^^,^^10^ Several different geographical methods – and combinations of such methods – can be used to generate a geographical sampling frame. For example, if the simultaneous export of a large number of points is not a concern, household structures can be digitized directly in Google Earth. Alternative software is available if researchers cannot obtain an ArcGIS license. For example, QGIS^11^ is open-source and includes a random selection tool that can be used to create a representative sample of households. Our approach – or a variation of it – can provide an accurate and apparently cost-effective sampling frame that has multiple potential applications in resource-poor settings (Box 1).

Box 1 **Summary of main lessons learnt**

- In the development of a geographical sampling frame, the use of Google Earth imagery and geographical software was an efficient alternative to the use of a demographic surveillance system.
- The use of a complete list of household coordinates reduced the time needed to locate households in the random sample.
- The approach that we used to generate a sampling frame appears accurate and cost-effective and has utility beyond morbidity studies, even in resource-poor settings.
